# Role of serum immunoglobulins for predicting sarcoidosis outcome: A cohort study

**DOI:** 10.1371/journal.pone.0193122

**Published:** 2018-04-11

**Authors:** Nicolas Belhomme, Stéphane Jouneau, Guillaume Bouzillé, Olivier Decaux, Mathieu Lederlin, Stéphanie Guillot, Antoinette Perlat, Patrick Jégo

**Affiliations:** 1 Internal Medicine Department, Rennes University Hospital, Rennes, France; 2 University of Rennes 1, Rennes, France; 3 Department of Respiratory Medicine, Rennes University Hospital, Rennes, France. University of Rennes 1, Rennes, France; 4 INSERM-IRSET UMR1085, Rennes, France; 5 INSERM, U1099, Rennes, France; 6 Université de Rennes 1, LTSI, Rennes, France; 7 CHU Rennes, CIC Inserm 1414, Rennes, France; 8 CHU Rennes, Centre de Données Cliniques, Rennes, France; 9 Department of Radiology, Rennes University Hospital, Rennes, France; 10 Department of Respiratory Physiology, Rennes University Hospital, Rennes, France; Ohio State University Wexner Medical Center, UNITED STATES

## Abstract

**Background:**

Sarcoidosis is a systemic granulomatous disease which carries variable outcomes. Serum protein electrophoresis is an easily accessible and routinely performed examination at diagnosis, in order to search for hypergammaglobulinemia, which is frequently found, and to rule out other granulomatous diseases such as common variable immunodeficiency. We aimed to assess the impact of baseline immunoglobulin level on the outcome of sarcoidosis.

**Methods:**

We conducted a retrospective cohort-study, at Rennes University Hospital, in which all newly diagnosed patients for whom a serum protein electrophoresis had been performed at baseline were enrolled, from 2006 to 2014. The main outcome was the need for corticosteroid treatment within 2 years from diagnosis, the secondary outcome was the occurrence of relapse among treated patients.

**Results:**

Eighty patients were included in the study, and 41.25% of them exhibited an elevated globulins rate. In univariate analysis, an elevated ACE level >70 U/l, Afro-Caribbean origin, and extra-pulmonary involvement, were associated with the need for corticosteroid treatment. In multivariate analysis, only ACE elevation (OR = 1.03, IC95% 1.01–1.05, p = 0.009) and extra-pulmonary involvement (OR = 5.8, IC95% 1.4–24, p = 0.015) were significant. Immunoglobulin level was not associated with the main outcome. Regarding the secondary outcome, none of the studied features were predictive of relapse among the 34 treated patients followed for two years.

**Conclusions:**

There was no relation between the immunoglobulin level at diagnosis and the evolution of sarcoidosis. An elevated ACE level and the presence of initial extra-pulmonary involvement were both associated with a more severe course of the disease necessitating a corticosteroid treatment.

## Introduction

Sarcoidosis is a systemic disease of unknown etiology, which is characterized by the development of non-necrotizing epithelioid granulomas in involved organs [1]. The course of the disease is unpredictable: spontaneous remission may occur while 20 to 70% of the patients will need a systemic therapy, for which oral corticosteroids are considered as the first-line option [1–3]. Relapse arises in 37 to 74% of treated patients [4]. Biologically, an elevated ACE is found in 60% of the patients [5]. Other parameters, such as interleukin-2 receptor, neopterin, or chitotriosidase, have been proposed for monitoring the activity of the disease, nevertheless their prognosis value needs to be confirmed, and their accessibility in routine practice is limited [6–8]. Hypergammaglobulinemia is frequently observed among sarcoidosis patients, but its relation to the disease’s course has not been established [9,10]. It has been suggested that elevated immunoglobulins or circulating immune-complex may be correlated to the disease’s course [11, 12], nonetheless no recent study has aimed to further explore this hypothesis to our knowledge [13]. As immunoglobulin level is highly influenced by many factors such as corticosteroid treatment or intercurrent infections, it is not appropriate for monitoring the disease’s activity. However, serum protein electrophoresis (SPE) is frequently performed at diagnosis, and easily accessible in routine practice. Hence, the aim of our study was to investigate the potential role of the immunoglobulin level at the time of diagnosis for predicting the disease’s course.

## Methods

### Study design

We conducted a retrospective cohort study in Rennes University Hospital, a tertiary care center referral for rare pulmonary diseases, between January, 1^st^ 2006 and December, 31^st^ 2014. All of the data used in this study existed prior to the study and the request for ethic approval, and was collected as part of standard medical procedure. The authors had access to the identity of included patients.

Ethics approval was received from the Rennes University Hospital ethics committee (number 16.65, 2016, June 6^th^). The need for consent was waived by the ethics committee for this particular study, and only the non-opposition of participating patients had to be collected. Therefore, all patients received a written information about the study and were given the possibility to decline to participate by completing and returning an opposition form which was specific to this particular study. Minors were not included. All patients newly diagnosed with sarcoidosis during the study period were included. Potential cases were retrieved from the institutional clinical data warehouse, eHOP software [14], and their medical records were individually reviewed. Sarcoidosis was diagnosed in accordance with the American Thoracic Society and World Association of Sarcoidosis and Other Granulomatous disorders (WASOG) criteria [2]. Histological confirmation was not needed for patients presenting with typical Löfgren’s syndrome [1,2]. The time of diagnosis was defined as the date of the first positive histopathological result or the date of the clinical diagnosis.

Inclusion required for a SPE to have been performed in our center within 3 months from diagnosis, before any systemic anti-sarcoidosis medication was started, and for the patients not to have been exposed to corticosteroids within a period of 4 month prior to SPE. Patients with chronic diseases that affect immunoglobulins amount and function (e.g primary immune deficiency, cirrhosis, hemopathy, HIV infection) were excluded. Patients were followed up for 2 years, in accordance with previous studies addressing the disease’s prognosis [15,16]. The primary outcome was the need for corticosteroid (CS) treatment. The decision to treat was taken according to guidelines [1,2,17]. The secondary outcome was the association between the baseline gammaglobulin level and the occurrence of relapse. Organ involvement was determined using the 2014 WASOG assessment instrument, and only involvements classified as “highly probable” or “at least probable” were considered [18,19]. Organ involvements were deemed extra-pulmonary if they did not involve lungs or mediastinal lymph nodes. Neither hypercalcemia, nor Löfgren’s syndrome or erythema nodosum were considered as extra-pulmonary involvement. Visceral involvement was defined by the involvement of the heart, liver, central nervous system, kidney or spleen. Relapse was defined by the necessity of escalating CS therapy due to progressive disease, or of resuming CS therapy after cessation [20].

**Fig 1:**
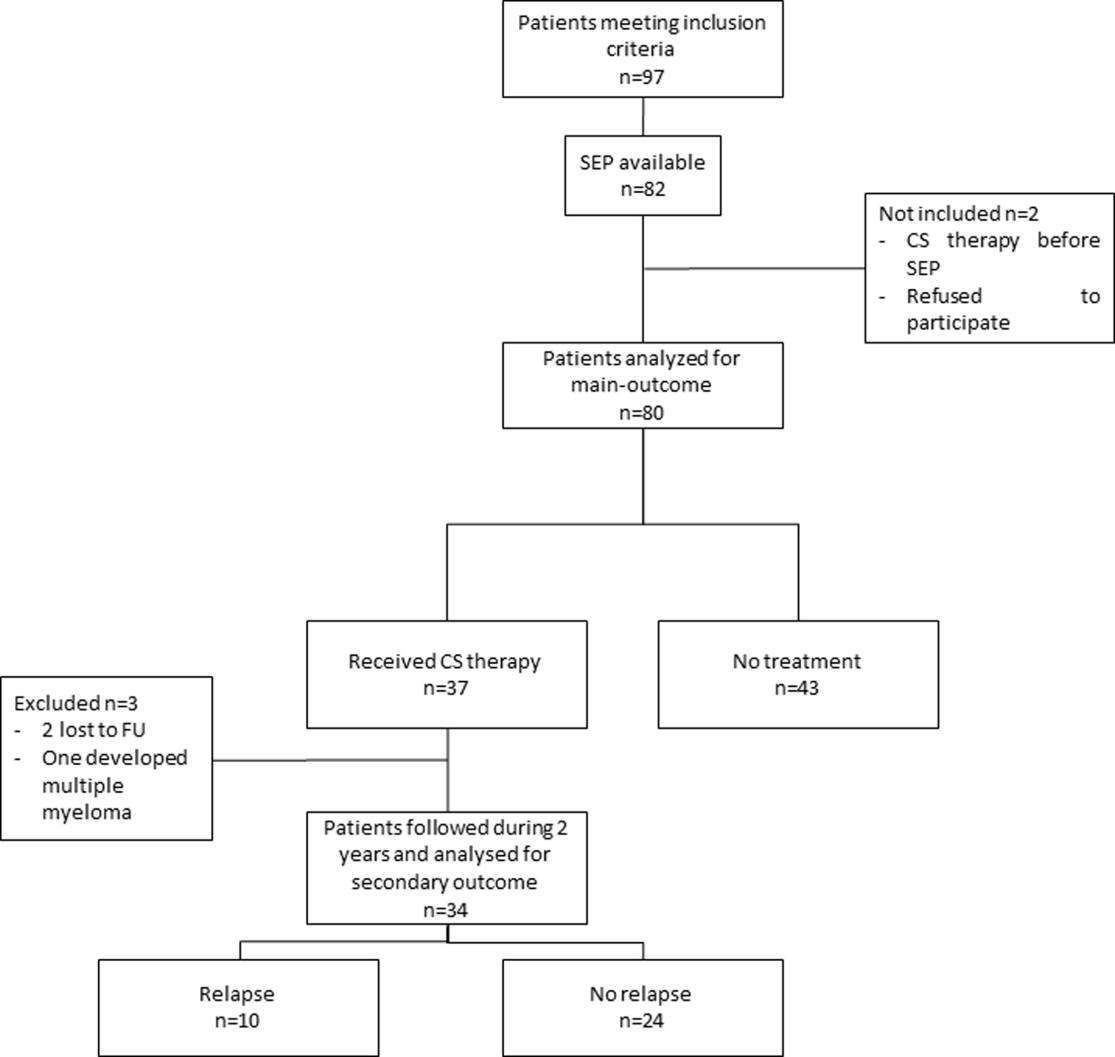
Study profile. CS = Corticosteroids; FU = Follow-Up; SPE = Serum Protein Electrophoresis.

Between January 1^st^ 2006 and December 31^st^ 2014, sarcoidosis was diagnosed according to WASOG criteria in 97 patients in our center. SPE had been performed at diagnosis in 82 of them. Among those, one had received corticosteroids within one month prior to diagnosis, and one refused to participate. Consequently, 80 patients were finally included in the analysis. Three patients were excluded during the follow-up period: 2 were lost to follow up, and one developed multiple myeloma requiring chemotherapy. All of these 3 patients were included in the analysis for main outcome, as the severity of their disease brought the need for CS treatment before they were excluded from the analysis. They were not analyzed for secondary outcome as they didn’t complete the follow up period (Fig 1).

### Demographics and clinical data collection

We gathered demographic variables (age, ethnicity, gender), clinical variables (histological confirmation, involvement of the organs and manifestations of the disease). Pulmonary function tests (Forced Expiratory Volume in on second-VEF_1_, Total Lung Capacity-TLC and Diffusing Capacity of the Lung for Carbon Monoxide-DLCO) were analyzed, and roentgenographic stage at baseline was retrieved from the patient’s records. Among biological variables, we collected Angiotensin converting Enzyme (ACE) (normal range:20–70 U/l), lymphocytes count (normal range: 1000–4000 cells/μl), gammaglobulin level (normal range: 8–13.5g/l), creatininemia (normal: 0.67–1.18 mg/dl), and calcemia (normal: 2.26–2.62 mmol/l). SPE were all performed in our center using a capillary electrophoresis (Capillarys 2 from Sebia Inc, Norcross, USA, with capillary Protein(e) 6 assay, ref 2003). Normal range was 8–13.5 g/l.

### Statistical analysis

Quantitative variables are given with their mean±SD or with their median and interquartile range (IQR) depending on their distribution. Comparison between quantitative variables were analyzed by conducting Student or Mann Whitney U test. The analysis of predictive factors associated with study outcomes were conducted by logistic regression using Firth method for correction of bias. Results are given with their Odds-Ratio (OR) and 95% confidence interval (CI95%). All variables were studied in univariate analysis. Factors for which the corresponding p-value was <0.10 were included in multivariate analysis and a stepwise selection procedure was applied to get the final models. Missing data were taken into account with the use of the multiple imputation by chained equations procedure. Five datasets were imputed to perform analyses. We followed Rubin’s rules to obtain the final results. The rates of missing data were in every case in the range of 0–6% (variables associated with the highest rates of missing data were TLC and DLCO with a rate of 6%).

In order to test specifically the prognostic value of serum immunoglobulin level, this variable was added separately into the logistic regression model, and the area under curve (AUC) of the two predictive models (without immunoglobulin level and after including immunoglobulin level) were compared, and the likelihood ratio was calculated. Analysis were conducted with R version 3.3.3. software.

## Results

### Characteristics of the study population

Mean age of the patients was 39.0±14.69 years, sex ratio (f/m) was 1.5. Regarding ethnicity, 69(86.25%) patients were Caucasian, 8 (10%)were Afro-Caribbean. Forty-four (55%) patients had an extra-pulmonary involvement at diagnosis. Chest X-ray showed a stage 1 in most of the patients (n = 38, 47.5%). Median immunoglobulin level was 12.90 g/l (10.17–14.57, extremes 8.1–35.9). Thirty-three (41.25%) patients presented a hypergammaglobulinemia >13.5g/l. A CS therapy was needed for 37 (46.25%) patients during the study period. Population characteristics are presented in Table 1.

**Table 1:** Patients characteristics.

**Patients characteristics**	
Sex ratio (f/m)	1.5 (48/32)
Age (mean±SD)	39±14.69
**Clinical features**	n(%)
Ethnicity	
Caucasian	72(90)
Caribbean	3(3.75)
African	5(6.25)
Histological confirmation	63(78.75)
Löfgren’ syndrome	26(32.5)
Extra-pulmonary involvement	44(55)
Periph. adenopathy	21(26.25)
Ocular	10(12.5)
Arthritis	9(11.25)
Cutaneous sarcoid	9(11.25)
ENT	8(10)
Liver	7(8.75)
Spleen	5(6.25)
Nervous System	3(3.75)
Muscles	2(2.5)
Heart	2(2.5)
Bones	1(1.25)
Respiratory symptoms	
Dyspnea	23(28.75)
Cough	19(23.75)
**Chest X-ray stage**	n(%)
Normal	9(11.25)
I	38(47.5)
II	29(36.25)
III	4(5)
IV	0
**Biological data**	Median (Q1-Q3)
Immunoglobulin level (g/l)	12.90(10.17–14.57)
Blood lymphocytes count (/μl)	1.21(0.87–1.58)
ACE (U/l) *	67(44–92.75)
Calcemia (mmol/l)	2.39(2.32–2.46)
Creatininemia (mg/dl)	0.82(0.68–0.93)
**Pulmonary function tests** (% Pred)	Median (Q1-Q3)
FEV_1_**	103.9(91.9–112.9)
TLC***	102(92.85–113.95)
DLCO***	70.20(62.65–80.90)
**Treatments received**	n(%)
Corticosteroids	37(46.25)
Hydroxychloroquine	3(3.75)
**Relapse**	10(12.5)

* Data available for 78 patients.

** Data available for 77 patients;

*** Data available for 75 patients.

ACE = Angiotensin Converting Enzyme; DLCO = Diffusion Capacity of Lungs for Carbon Monoxide; ENT = Ear, Nose and Throat; FEV_1_ = Forced Expired Volume in one second; TLC = Total Lung Capacity.

### Relation of immunoglobulin level to individual features

Median immunoglobulin level was significantly higher in Afro-Caribbean patients (19.45 versus 12.56 g/l, p<0.001) and in patients presenting an extra-pulmonary involvement (13.65 versus 11.85, p = 0.016). Patients presenting a Löfgren’s syndrome had a lower immunoglobulin level (10.90 versus 13.55, p = 0.011). We found a relation neither between immunoglobulin level and ACE level (p = 0.11), nor with a decrease in pulmonary function test parameters. Patients who necessitated a corticosteroid treatment during the study period and those who presented a relapse had a significantly higher immunoglobulin level (13.60 versus 12.40 g/l, p = 0.036 and 15.15 versus 12.53g/l, p = 0.014, respectively) (Table 2).

**Table 2:** Relation between immunoglobulins and individual characteristics.

Variables		Median value of immunoglobulins (Median Q1-Q3)	p- value
Age			
>40		13.0 (10.2–14.5)	
≤40		12.7 (10.2–14.6)	0.93
Gender			
Female n = 48		12.90 (10.25–14.57)	
Male n = 32		13.05 (10.17–14.50)	0.976
Ethnicity			
Afro-Caribbean n = 8		19.45 (14.85–25.27)	
Other n = 72		12.56 (10.07–14.05)	**<0.001**
Chest X-ray stage			
0 or 1 n = 47		12.90 (10.25–14.65)	
>1 n = 33		13.00 (9.90–14.20)	0.957
Extra-pulmonary involvement			
No n = 36		11.85 (9.83–13.60)	
Yes n = 44		13.65 (10.70–15.43)	**0.016**
Löfgren’s syndrome			
Yes n = 26		10.90 (9.98–12.90)	
No n = 54		13.55 (10.33–15.47)	**0.011**
ACE*			
>70 U/l		13.7 (10.4–15.5)	
≤70 U/l		11.6 (9.7–13.5)	0.11
Pulmonary function test			
FEV_1_**	≥80%	12.9 (10.1–14.5)	
	<80%	13.3 (10.3–14.2)	0.83
TLC***	≥80%	12.9 (10.1–14.5)	
	<80%	24.8 (19.3–30.4)	0.11
DLCO***	≥75%	12.5 (9.7–13.8)	
	<75%	13.2 (10.4–14.6)	0.23
CS therapy			
No (n = 43)		12.40 (9.95–13.90)	
Yes (n = 37)		13.60 (10.55–15.45)	**0.036**
Relapse			
No (n = 24)		12.53 (10.15–13.95)	
Yes (n = 10)		15.15 (13.82–16.82)	**0.014**

* Data available for 78 patients

** Data available for 77 patients

*** Data available for 75 patients.

ACE = Angiotensin Converting Enzyme; DLCO = Diffusion Capacity of Lungs for Carbon Monoxide; FEV_1_ = Forced Expired Volume in one second; TLC = Total Lung Capacity.

### Results for main outcome

In univariate analysis, neither the immunoglobulin level at diagnosis, nor hypergammaglobulinemia, were associated with CS treatment (OR = 1.1, CI95% 1–1.3, p = 0.059 and OR = 2, CI95% 0.79–5.2, p = 0.137, respectively). Factors that were predictive of the need for treatment were the Afro-Caribbean origin (OR = 24, CI95% 1–554, p = 0.049), an ACE level >70 U/L (OR = 6.2, CI95% 2.2–17, p<0.001), extra-pulmonary involvement (OR = 6.7, CI95% = 2.4–19, p<0.001) including cutaneous localization (OR = 7.7, CI95% = 1.1–55, p = 0.041), ENT (OR = 7.7, IC95% = 1.1–55, p = 0.041) and lymph nodes involvement (OR = 5.6, CI95% 1.7–18, p = 0.004). Löfgren’s syndrome was associated with treatment non-necessity (OR = 0.097, CI95% 0.027–0.35, p<0.001). The results of the univariate analysis are shown in Table 3. In multivariate analysis, both extra-pulmonary involvement (OR = 5.8, CI95% 1.4–24, p = 0.0152) and an elevated ACE (OR = 1.03, CI95% = 1.01–1.05, p = 0.009, meaning that each elevation of one unit of ACE increased the probability for treatment of 3%) were predictive of the need for CS (Table 4, Model 1). These findings remained unchanged after including the immunoglobulin level into the logistic regression model (Table 4, Model 2). The likelihood ratio for the two models was 1, reflecting the absence of influence of the immunoglobulin level on the main outcome.

**Table 3:** Features associated with corticosteroid therapy and relapse in univariate analysis.

	Main outcome(n = 80)			Secondary outcome (n = 34)		
Variables	OR	IC95%	p	OR	CI95.	p
Age	1	0.98–1.04	0.659	0.97	0.9–1	0.315
Gender (female)	1.8	0.71–4.7	0.212	0.69	0.15–3.2	0.629
Ethnicity	24	1–554	**0.049**	1	0.16–6.8	0.964
X-ray stage >1	2.5	0.98–6.6	0.055	0.85	0.19–3.9	0.831
Immunoglobulin level	1.1	0.99–1.3	0.059	1.1	0.96–1.4	0.122
Immunoglobulins >13.5g/l	2	0.79–5.2	0.137	5.5	0.97–32	0.054
Lymphocytes count	0.96	0.55–1.7	0.886	0.67	0.21–2.2	0.494
ACE	1	1–1.05	**0.002**	1	0.99–1.03	0.143
ACE >70U/l	6.2	2.2–17	**<0.001**	3.3	0.41–26	0.256
Calcemia	2.9	0.86–10	0.084	0.97	0.59–1.6	0.892
Creatininemia	5.1	0.49–52	0.169	0.54	0.022–14	0.702
Dyspnea	1.8	0.64–4.9	0.264	4.1	0.82–21	0.083
Cough	0.42	0.13–1.3	0.141	1.8	0.24–14	0.557
Löfgren’s syndrome	0.09	0.03–0.3	**<0.001**	0.29	0.0076–11	0.497
Extra-pulmonary involvement	6.7	2.4–19	**<0.001**	9	0.34–242	0.183
Visceral involvement	6.2	1.3–30	**0.023**	4.6	0.84–25	0.0776
Arthritis	0.8	0.2–3.6	0.743	0.29	0.0076–11	0.497
Cutaneous	7.7	1.1–55	**0.041**	2.1	0.35–13	0.397
Ocular	1.6	0.39–6.8	0.492	1.8	0.24–14	0.557
ENT	7.7	1.1–55	**0.041**	1	0.16–6.8	0.964
Periph. adenopathy	5.6	1.7–18	**0.004**	2.4	0.5–11	0.268
Spleen	4.2	0.51–35	0.181	7.3	0.67–80	0.1
Liver	6.5	0.88–47	0.0657	6.2	0.87–45	0.068
Nervous system	2.2	0.19–25	0.524	2.5	0.12–49	0.542
TLC	0.97	0.93–1	0.044	0.98	0.92–1.1	0.631
FEV_1_	0.97	0.94–1	0.115	1	0.96–1.1	0.667
DLCO	0.95	0.91–1	0.033	0.99	0.93–1.1	0.834

ACE = Angiotensin Converting Enzyme; DLCO = Diffusion Capacity of Lungs for Carbon Monoxide; FEV_1_ = Forced Expired Volume in one second; TLC = Total Lung Capacity.

**Table 4:** Features associated with corticosteroid therapy in multivariate analysis.

Variables	Model 1			Model 2		
	OR	CI95%	p	OR	CI95%	P
Ethnicity	20	0.6–659	0.093	19	0.61–618	0.092
Extra-pulmonary involvement	5.8	1.4–24	**0.015**	5.6	1.4–22	**0.016**
Chest X-ray stage	4	0.99–16	0.052	3.8	0.97–15	0.056
ACE	1.03	1.01–1.05	**0.009**	1.03	1.006–1.05	**0.010**
Immunoglobulin level	-	-	-	0.97	0.8–1.2	0.791

ACE = Angiotensin Converting Enzyme.

### Results for secondary outcome

Relapse was analyzed in the 34 patients who received CS and were followed for two years.Their characteristics are presented in an additional table (S1 Table). A relapse occurred in 10 of them. The duration of exposure to the secondary outcome was comparable (22 months from start of treatment to end of study in patients with relapse versus 20 months for non-relapsing patients, p = 0.24). In univariate analysis, we didn’t find any factors that predict relapse. Particularly, neither the immunoglobulin level (OR = 1.1, CI 95% 0.96–1.4, p = 0.122), nor hypergammaglobulinemia (OR = 5.5, CI95% 0.97–32, p = 0.054) were associated with relapse (Table 3). In multivariate analysis, variables which were retained in the model were dyspnea (OR = 5.1, CI95% 0.85–31, p = 0.07) and visceral involvement (OR = 5.7, CI95% 0.87–38, p = 0.06), and none were significant. Once added into the model, the immunoglobulin level was also not significant (OR = 1.1, CI95% 0.91–1.4, p = 0.326). However, it resulted in an improvement of the relapse predicting value of the model. Indeed, the likelihood ratio of the two models favored the second one (p = 0.012), and the derivation area under curve was 0.77 (CI95%: 0.56–0.97) for model 2, comprising immunoglobulins, whereas it was 0.63 (CI95%: 0.41–0.85, non-significant) for model 1.

## Discussion

At the end, although the patients who required CS therapy and those who experienced a relapse had a higher baseline immunoglobulin level, our results show that such a parameter is not of interest for predicting the need for CS therapy during the first 2 years following diagnosis. Among study parameters, only ACE and extra-pulmonary involvement were found to be associated with the main outcome. In our study, 41.25% of the patients had a hypergammaglobulinemia at diagnosis, an intermediate value compared to those found in other studies [11,21]. Patients from Afro-Caribbean ethnicity had a higher rate of serum immunoglobulin compared to Caucasians (19.45 versus 12.56 g/l, p<0.001). This finding is in line with the results of Kataria and Stilzbach [11,22]. Group comparison analysis revealed that patients undergoing corticosteroid therapy had a higher immunoglobulin level than the non-treated patients (13.6 versus 12.4 g/l, p = 0.036). Treated patients who experienced a relapse had a higher rate of immunoglobulin as well (15.15 versus 12.53 g/l, p = 0.014). Multiple logistic regression showed that the serum immunoglobulin level was associated neither with disease severity as assessed by the need for CS therapy, nor with the occurrence of relapse. In multivariate analysis, extra-pulmonary involvement at diagnosis was associated with CS therapy (OR = 5.8, CI95% 1.4–24, p = 0.015). We used a stepwise selection procedure to identify the final predictors. This procedure is known to potentially fail at identifying the true predictors. However, our findings are consistent with previous studies, which showed that initial extra-pulmonary involvement entails a worsening in the disease’s course resulting in additional organs involvements [16,23].

An elevated ACE was also predictive of the need for treatment, and this result is in line with many studies which have discussed the potential role of ACE for monitoring disease activity [24,25]. Nevertheless, whether ACE is a reliable marker of disease activity remains very controversial. Moreover, its reliability suffers from significant individual variations even in healthy subjects, mainly due to genetic polymorphism: thus, we should remain very cautious regarding its interpretation [26–28]. Finally, we observed a clear-although not significant-trend on the association between the Afro-Caribbean ethnicity and the need for treatment. As already shown in previous studies, black ethnicity is associated with more severe forms of the disease [29–32], to a more frequent need for systemic therapy [30]. and to a higher mortality [33,34]. Although there was also no association between black ethnicity and treatment in a study conducted by Baughman [35], the non-significance of this result in the present study may be due to insufficient power as few black patients were included.

There are several limitations to our study. First of all, the number of patients (n = 80) may have impaired the statistical power of our analysis. Indeed, our inclusion criteria were very restrictive, and only the patients whose diagnosis was histologically confirmed or who presented a typical Löfgren’s syndrome were considered, in accordance to international WASOG criteria [1,2]. Nonetheless, these criteria may be too restrictive: it has been shown that a typical presentation on chest CT-scan, and hyperlymphocytosis with a CD4/CD8 ratio greater than 3.5 on broncho-alveolar lavage, combined with the exclusion of other possible diagnosis, are highly predictive of sarcoidosis [36,37]. Then, the possibility of retaining the diagnosis in such patients should be discussed: we may consider the sarcoidosis as “probable” in cases fulfilling these criteria, whereas it would be classified as “certain” once histological proof is obtained. The secondary outcome is limited by the few relapses observed: indeed, only 10 relapses occurred, concerning 29.4% of the 34 treated patients followed-up for 2 years. This is principally due to the definition employed: we made the choice to consider the relapse only among the patients who received or had received a treatment. As a consensual international definition of relapse is lacking, we aimed to avoid confusion between relapse and disease progression, which could be defined by the need of introducing a treatment due to disease aggravation after a period of stability [17,20]. Including this feature of the disease into the analysis would have biased the main outcome. Furthermore, CS therapy on its own may be associated with a higher risk of relapse [15,17,38], and limiting the assessment of relapse to the treated patients rather than assessing the whole population permitted to prevent this interaction bias. Another explication to the low number of relapses is the duration of follow-up, limited to 2 years. Thereby, only early relapses were studied. However, the risk of relapse lasts for many years over the disease’s course, and our results do not reflect the global risk of relapse of sarcoidosis, as it was not our purpose [3,4,17].

Moreover, the risk of relapse is highest in the year following therapy disruption: in our study 20 out of the 34 treated patients were still receiving therapy at the end of follow up, thus carrying a lower potentiality of relapse [4]. On top of that, the risk of relapse is higher in black patients, who were a small number in our cohort. Finally, the relapse rate observed in our study is comparable to the study of Rizzato et al., which was also conducted in an essentially Caucasian population [4]. It is worth noting that the inclusion of the immunoglobulin level variable improved substantially the predicting capacity for relapse of the model, which implies that immunoglobulin level at diagnosis may be of interest for predicting relapse. This hypothesis needs to be further investigated in larger studies.

## Conclusions

In this cohort study including 80 patients followed for two years after sarcoidosis was diagnosed, we didn’t find an association between the immunoglobulin level at baseline and the need for CS therapy or the occurrence of relapse. Nevertheless, the patients requiring a treatment and those who experienced a relapse had a higher level of immunoglobulin, as well as Afro-Caribbean patients. The immunoglobulin level statistically improved the capacity of the model to predict relapse, bringing the need for further larger studies to confirm its potential prognosis value. Furthermore, a significant association was found between initial extra-pulmonary involvement or an elevated ACE and the need for CS therapy.

## Supporting information

S1 Table Characteristics of the 34 patients who received a corticosteroid therapy and who completed the 2 years follow-up period. *Data available for 33/34 patients; **Data available for 32/34 patients.
(DOCX)
