# Supplementary material for: Role of serum immunoglobulins for predicting sarcoidosis outcome: A cohort study
Source: PLoS One. 2018 Apr 11;13(4):e0193122. doi: 10.1371/journal.pone.0193122 (PMC5894960; doi:10.1371/journal.pone.0193122)
Supplement: S1 Table — *Data available for 33/34 patients; **Data available for 32/34 patients. (DOCX) [file pone.0193122.s001.docx]

| Patients characteristics (n=34) | n(%) |
| --- | --- |
| Sex ratio (f/m) | 1.13 (18/16) |
| Age (mean±SD) | 39.18±13.27 |
| Clinical features | n(%) |
| Ethnicity |  |
| - Caucasian - Caribbean - African | 27(79.41)  2(5.88)  5(14.71) |
| Histological confirmation | 33(97.06) |
| Löfgren’ syndrome | 3(8.82) |
| Extra-pulmonary involvement | 27(79.41) |
| - Periph. adenopathy - Ocular - Arthritis - Cutaneous sarcoid - ENT - Liver - Spleen - Nervous System - Muscles - Heart - Bones | 15(44.11)  5(14.71)  3(8.82)  7(20.59)  7(20.59)  6(17.64)  4(11.76)  2(5.88)  2(5.88)  2(5.88)  1(2.94) |
| Respiratory symptoms |  |
| - Dyspnea - Cough | 12(35.29)  5(14.71) |
| Chest X-ray stage | n(%) |
| - Normal - I - II - III - IV | 2(5.88)  14(41.18)  15(44.18)  3(8.82)  0 |
| Biological data | Median (Q1-Q3) |
| - Immunoglobulin level (g/l) - Blood lymphocytes count (/µl) - ACE (U/l) * - Calcemia (mmol/l) - Creatininémia (mg/dl) | 13.6(10.55-15.45)  1.04(0.81-1.53)  88.5(70.25-117)  2.42(2.34-2.49)  0.87(0.69-0.99) |
| Pulmonary function tests (% Pred) | Median (Q1-Q3) |
| - FEV_1_* - TLC** - DLCO** | 99.5(88.2-110.6)  100.2(92.3-104.5)  65.25(60.7-73.1) |
| Treatments received | n(%) |
| - Corticosteroids - Hydroxychloroquine - Immunosuppressive therapy | 34(100)  1(2.94)  0 |
| Relapse | 10(29.4) |

S1 Table : characteristics of the 34 patients who received a corticosteroid therapy and who completed the 2 years follow-up period.

*Data available for 33/34 patients

**Data available for 32/34 patients
